# Conditional relative survival among patients with nodular lymphocyte-predominant Hodgkin lymphoma in the Netherlands

**DOI:** 10.1038/s41408-021-00482-8

**Published:** 2021-05-11

**Authors:** Hidde L. A. Posthuma, Josée M. Zijlstra, Otto Visser, Marie José Kersten, Pieternella J. Lugtenburg, Avinash G. Dinmohamed

**Affiliations:** 1 Department of Internal Medicine, Noordwest Ziekenhuisgroep, Alkmaar, The Netherlands; 2 Amsterdam UMC, Vrije Universiteit Amsterdam, Department of Hematology, Cancer Center Amsterdam, Amsterdam, The Netherlands; 3 Department of Registration, Netherlands Comprehensive Cancer Organisation (IKNL), Utrecht, The Netherlands; 4 Amsterdam UMC, University of Amsterdam, Department of Hematology, Cancer Center Amsterdam, LYMMCARE (Lymphoma and Myeloma Center Amsterdam), Amsterdam, The Netherlands; 5 Erasmus MC Cancer Institute, University Medical Center Rotterdam, Department of Hematology, Rotterdam, The Netherlands; 6 Department of Research and Development, Netherlands Comprehensive Cancer Organisation (IKNL), Utrecht, The Netherlands; 7 Erasmus MC, University Medical Center Rotterdam, Department of Public Health, Rotterdam, The Netherlands

**Keywords:** Hodgkin lymphoma, Lymphoma, Epidemiology, Risk factors, Cancer epidemiology

Dear Editor,

Nodular lymphocyte-predominant Hodgkin lymphoma (NLPHL) is a rare and clinicopathologically distinct entity within the spectrum of Hodgkin lymphomas^1,2^. Population-based studies in NLPHL have provided grounds for optimism that most patients with NLPHL have survival expectations that approximate those of the general population^3–7^.

Although excess mortality in NLPHL is generally low, patients with older age and advanced disease do experience excess mortality within the first few years after diagnosis^4–6,8^. Therefore, the available statistics on NLPHL survival—which are all measured from diagnosis or the start of treatment—might be somewhat pessimistic about informing long-term NLPHL survivors about their prognosis over time. This problem can be tackled by estimating the survival of patients who survive a specified time since diagnosis (i.e., conditional survival). Estimating conditional survival accounts for time-dependent mortality rates, which are generally increased in the first few years after diagnosis. Conditional survival estimates become even more valuable when they are corrected for the life expectancy in the general population; that is, conditional relative survival (CRS).

To our knowledge, data on CRS in NLPHL are lacking. These data could help to inform patients and physicians about the changes in NLPHL prognosis over time and the intensity of surveillance and follow-up activities based on the patients’ risk of mortality over time. In this population-based study, we assessed 5-year relative survival (RS) at diagnosis and 5-year CRS for each following year after diagnosis up to 10 years post-diagnosis among adult patients diagnosed with NLPHL in the Netherlands.

Established in 1989, the Netherlands Cancer Registry (NCR) has national coverage of at least 95% of all newly diagnosed malignancies in the Netherlands^9^. We identified all adult (≥18 years) patients diagnosed with NLPHL during 1993–2018—with survival follow-up through December 31, 2019—from the NCR. The International Classification of Diseases for Oncology morphology code 9659 was used to select patients with NLPHL from the NCR. That code was introduced in 1993 based on the REAL classification introduced in that same year^10^. Therefore, patient selection was restricted to those diagnosed as of 1993. The Privacy Review Board of the NCR approved the use of anonymous data for this study.

We calculated 5-year RS at diagnosis and 5-year CRS for each additional year survived up to 10 years post-diagnosis. RS is a measure that approximates disease-specific survival in the absence of information on the cause of death, which is not ascertained in the NCR. RS is calculated as the ratio of the patients’ overall survival (OS) to the expected survival of equivalent groups from the general population—matched to the patients by age, sex, and calendar year^11^. The Ederer II method was used to estimate the expected survival using Dutch population life tables^12^.

RS was estimated using a hybrid approach—which is conceptually similar to the approach used to estimate the life expectancy at birth—to predict up-to-date survival statistics in an unbiased manner when incidence data lag behind mortality statistics^13^. The hybrid approach was applied for patients diagnosed during 1993–2018 who were alive at some point during the follow-up interval 2007–2019, resulting in 15 years of post-diagnostic follow-up information. Supplementary Fig. 1 illustrates how the survival data were structured under the hybrid approach. The estimates produced by the hybrid approach can be interpreted as the predicted survival probabilities for patients diagnosed during 2007–2019. Further details about this approach are provided in the Supplementary Information.

RS was computed for the overall cohort and by sex and age (18–44 and ≥45 years) and disease stage at diagnosis (I–II and III–IV). The age cutoff was based on the International Prognostic Score^14^. Excess mortality is considered minimal when RS exceeds 95%^15^. Differences in CRS estimates between subgroups were considered statistically significant when the 95% confidence intervals (CIs) did not overlap. Analyses were performed using STATA Statistical Software version 16.1 (StataCorp, College Station, TX).

A total of 747 patients (median age 44 years; 51% aged 18–44 years; 74% males; and 70% stage I–II disease) were diagnosed with NLPHL in the Netherlands between 1993 and 2018 (Table 1). The number of patients at risk under the hybrid approach at diagnosis and 5- and 10-years post-diagnosis—along with the 5-year CRS at these time points—are summarized in Table 1 according to baseline characteristics. Figure 1 shows a graphical presentation of 5-year CRS up to ten years post-diagnosis for the overall cohort and by sex, age, and stage.

**Table 1:** Five-year relative survival at diagnosis and 5- and 10-years post-diagnosis among adult patients with nodular lymphocyte‐predominant Hodgkin lymphoma in the Netherlands according to baseline characteristics, 1993–2018.

Characteristics	No. of patients at diagnosis		No. of patients at risk under the hybrid approach after x year			Conditional 5-year relative survival (95% CI)		
	*N*	(%)	0	5	10	At diagnosis	At 5 years	At 10 years
Total no. of patients	747	–	729	475	292	93 (90–96)	99 (96–101)	93 (87–96)
*Sex*								
Male	555	(74)	542	355	226	93 (90–96)	99 (96–102)	93 (88–98)
Female	192	(26)	187	120	66	95 (90–100)	99 (94–104)	90 (80–102)
*Age at diagnosis*								
18–44 years	379	(51)	372	274	190	98 (96–100)	100 (99–101)	96 (92–100)
≥45 years	368	(49)	357	201	102	89 (85–94)	97 (91–103)	86 (75–98)
*Stage at diagnosis*								
I–II	526	(70)	515	362	237	98 (95–100)	101 (99–103)	94 (89–99)
III–IV	206	(28)	201	102	47	85 (79–92)	92 (83–101)	88 (75–103)
Unknown	15	(2)	13	11	8	─^a^	─^a^	─^a^

*CI* confidence interval.

^a^Survival was not calculated due to the very small number of patients.

**Fig. 1:**
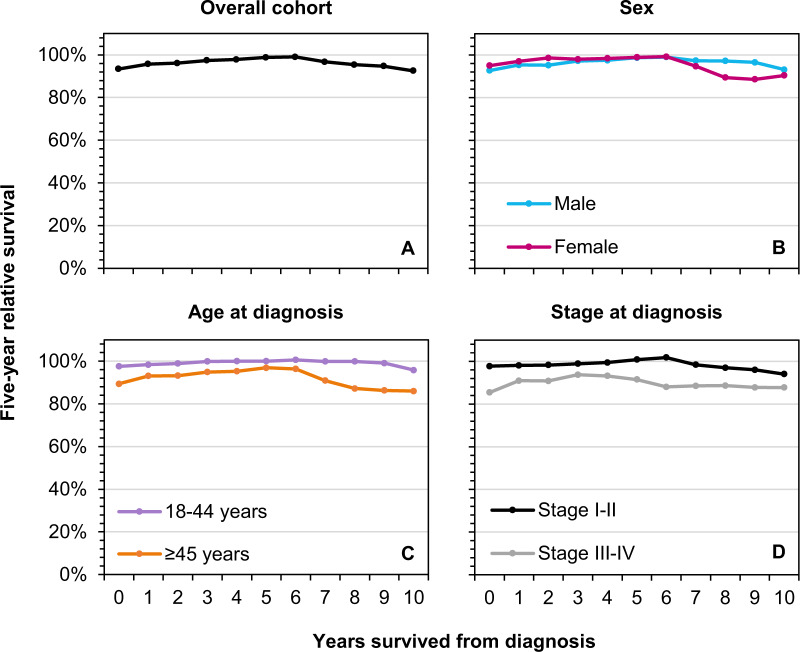
Conditional 5-year relative survival up to 10 years post-diagnosis among adult patients with nodular lymphocyte-predominant Hodgkin lymphoma. Conditional 5-year relative survival up to 10 years post-diagnosis among adult patients with nodular lymphocyte-predominant Hodgkin lymphoma in the Netherlands according to (**A**) the overall cohort and by (**B**) sex, (**C**) age at diagnosis, and (**D**) stage at diagnosis.

Overall, 5-year RS at diagnosis was 93% (Fig. 1A). Five-year CRS for the overall cohort exceeded 95%—which reflects minimal excess mortality—at 1-year post-diagnosis. However, it became slightly less than 95% at 9 years post-diagnosis, indicating small excess mortality. No significant sex-related survival differences were observed throughout the follow-up period (Fig. 1B). The initial survival difference between the age (Fig. 1C) and stage groups (Fig. 1D) mostly diminished after 1-year post-diagnosis. Encouragingly, patients aged 18–44 years and those with stage I–II disease had 5-year CRS exceeding 95% throughout virtually the entire follow-up period. Minimal excess mortality was objectified among patients aged ≥45 years after 5 years post-diagnosis. However, 5-year CRS declined below 95% after 7 years post-diagnosis. Of note, 5-year CRS among patients with stage III–IV disease did not exceed 95% throughout the follow-up period.

The information from this first study on CRS in NLPHL provides a better appreciation of the prognosis with each additional year survived from diagnosis. The overall prognosis of NLPHL can be regarded as favorable.

We show that the well-established prognostic effect of older age and advanced stage mostly diminished with additional years survived after diagnosis^3,4,6^. This finding can be used to reassure most NLPHL survivors who were diagnosed with these adverse risk factors.

Another key finding of this study was that patients aged 18–44 years and those with stage I–II disease had 5-year CRS that virtually remained above 95% throughout the 15 years of post-diagnostic follow-up. This finding indicates that these patients have survival expectations similar to comparable groups from the general population. On the other hand—while the prognostic effect of stage largely diminished over time—5-year CRS did not exceed 95% throughout the follow-up period among patients with stage III–IV disease and remained around 90%, suggesting that these patients continued to endure small excess mortality over time. However, this finding should be interpreted in light of the wideness of the 95% CIs, which is related to the comparatively small number of patients in the group with stage III–IV disease. Nevertheless, the overall short- and long-term excess mortality encountered in NLPHL may be a consequence of treatment-related sequelae such as secondary malignancies, late recurrent disease or transformation to a high-grade lymphoma.

We demonstrated no prognostic effect of sex, which is congruent with some^6^—but not all—studies^5^. This finding could, in part, be related to the use of RS, instead of OS, in this study.

Limitations of the study include the lack of central pathology review and information on prognostic factors other than age and stage, detailed treatment strategies, and transformation and relapse rates. Nevertheless, our study provides patients with NLPHL and their physicians with valuable information about NLPHL prognosis during follow-up, which, in turn, can be used in concert with information on other risk factors to tailor surveillance and follow-up activities.

## Supplementary information

CRS in NLPHL in NL - Supplemental Information
