# Supplementary material for: Conditional relative survival among patients with nodular lymphocyte-predominant Hodgkin lymphoma in the Netherlands
Source: Blood Cancer J. 2021 May 11;11(5):87. doi: 10.1038/s41408-021-00482-8 (PMC8113262; doi:10.1038/s41408-021-00482-8)
Supplement: Supplementary file 1 — CRS in NLPHL in NL - Supplemental Information [file 41408_2021_482_MOESM1_ESM.docx]

**SUPPLEMENTAL INFORMATION**

**Title**

Conditional relative survival among patients with nodular lymphocyte-predominant Hodgkin lymphoma in the Netherlands

**Authors and affiliation**

Hidde L.A. Posthuma,^1,2^ Josée M. Zijlstra,^2^ Otto Visser,^3^ Marie José Kersten,^4^ Pieternella J. Lugtenburg,^5^ Avinash G. Dinmohamed^2,4,6,7^

^1^Department of Internal Medicine, Noordwest Ziekenhuisgroep, Alkmaar, The Netherlands; ^2^Amsterdam UMC, Vrije Universiteit Amsterdam, Department of Hematology, Cancer Center Amsterdam, Amsterdam, The Netherlands; ^3^Department of Registration, Netherlands Comprehensive Cancer Organisation (IKNL), Utrecht, The Netherlands; ^4^Amsterdam UMC, University of Amsterdam, Department of Hematology, Cancer Center Amsterdam, LYMMCARE (Lymphoma and Myeloma Center Amsterdam), Amsterdam, The Netherlands; ^5^Erasmus MC Cancer Institute, University Medical Center Rotterdam, Department of Hematology, Rotterdam, The Netherlands; ^6^Department of Research and Development, Netherlands Comprehensive Cancer Organisation (IKNL), Utrecht, The Netherlands; ^7^Erasmus MC, University Medical Center Rotterdam, Department of Public Health, Rotterdam, The Netherlands

**Supplemental methods**

***The hybrid approach***

Relative survival was estimated using the hybrid approach. The hybrid approach—which was described and empirically validated by Brenner and Rachet—is a modification of the period approach.(1) It incorporates both elements of the classical cohort and period approach. The hybrid approach enables unbiased estimation of up-to-date survival in situations where mortality data are more up-to-date than incidence data. For the current study, incidence data were available up to the year 2018, whereas follow-up for survival up to the year 2019. Therefore, the hybrid approach was the most appropriate approach to derive up-to-date survival for the current study.

The period approach incorporates the survival experience of patients during a specified period by left-truncating patients’ survival at the beginning of the period of interest, in addition to right-censoring patients’ survival at its end.(2) However, the use of the period approach requires that both incidence and mortality data are available for the most recent year within the period of interest. Therefore, to use the period approach in situations where mortality data are more up-to-date than incidence data, it should be combined with elements of the classic cohort approach.(3) For the current study, we considered the follow-up interval 2007-2019. More specifically, the cohort-based 1-year survival probability of patients diagnosed during 2007-2018 was combined with the period-based survival probabilities for the second to fifteenth years of follow-up among patients diagnosed during 1993-2018 who were alive at some point during the interval 2008-2019. The manner how we structured the survival data under the hybrid approach resulted in 15 years of post-diagnostic follow-up information to compute the conditional 5-year relative up to ten years post-diagnosis (Supplemental Figure 1). Supplemental Figures 2 depicts the survival time used—according to the years of diagnosis and years of follow-up—in the calculations of the 5-year relative survival at diagnosis and the conditional 5-year relative survival up to ten years post-diagnosis. In brief, five-year relative survival at diagnosis and the conditional five-year relative survival at one, two, three, five, and ten years post-diagnosis was considered through the survival experience of patients diagnosed during 2003-2008, 2002-2018, 2001-2017, 2000-2016, 1998-2014, and 1993-2009, respectively (Supplemental Figures 2 and Supplemental Table 2).

**Supplemental figure legends**

**Supplemental Figure S1.** Structure of the survival data used under the hybrid approach. The numbers within the cells denote the minimal years of follow-up (column) since diagnosis (row). For the computation of 5-year relative survival at diagnosis, and every additional year survived up to years post-diagnosis (that is, the conditional 5-year relative survival), the survival data in the grey shaded proportions were used.

**Supplemental Figure S2.** Survival data used in the calculation of 5-year relative survival at diagnosis (A) and after one (B), two (C), three (D), four (E), five (F), six (G), seven (H), eight (I), nine (J) and ten years post-diagnosis (K). The numbers within the cells denote the minimal years of follow-up (column) since diagnosis (row). The gray shaded proportions denote the survival data used in the calculation of 5-year relative survival.

**Supplemental Figure 1**

|  |  | **Year of follow-up** | | | | | | | | | | | | | | | | | | | | | | | | | | |  |  |
| --- | --- | --- | --- | --- | --- | --- | --- | --- | --- | --- | --- | --- | --- | --- | --- | --- | --- | --- | --- | --- | --- | --- | --- | --- | --- | --- | --- | --- | --- | --- |
|  |  | 1993 | 1994 | 1995 | 1996 | 1997 | 1998 | 1999 | 2000 | 2001 | 2002 | 2003 | 2004 | 2005 | 2006 | 2007 | 2008 | 2009 | 2010 | 2011 | 2012 | 2013 | 2014 | 2015 | 2016 | 2017 | 2018 | 2019 |  |  |
| **Year of diagnosis** | 1993 | 0 | 1 | 2 | 3 | 4 | 5 | 6 | 7 | 8 | 9 | 10 | 11 | 12 | 13 | 14 | 15 | 16 | 17 | 18 | 19 | 20 | 21 | 22 | 23 | 24 | 25 | 26 | 1993 | **Year of diagnosis** |
|  | 1994 |  | 0 | 1 | 2 | 3 | 4 | 5 | 6 | 7 | 8 | 9 | 10 | 11 | 12 | 13 | 14 | 15 | 16 | 17 | 18 | 19 | 20 | 21 | 22 | 23 | 24 | 25 | 1994 |  |
|  | 1995 |  |  | 0 | 1 | 2 | 3 | 4 | 5 | 6 | 7 | 8 | 9 | 10 | 11 | 12 | 13 | 14 | 15 | 16 | 17 | 18 | 19 | 20 | 21 | 22 | 23 | 24 | 1995 |  |
|  | 1996 |  |  |  | 0 | 1 | 2 | 3 | 4 | 5 | 6 | 7 | 8 | 9 | 10 | 11 | 12 | 13 | 14 | 15 | 16 | 17 | 18 | 19 | 20 | 21 | 22 | 23 | 1996 |  |
|  | 1997 |  |  |  |  | 0 | 1 | 2 | 3 | 4 | 5 | 6 | 7 | 8 | 9 | 10 | 11 | 12 | 13 | 14 | 15 | 16 | 17 | 18 | 19 | 20 | 21 | 22 | 1997 |  |
|  | 1998 |  |  |  |  |  | 0 | 1 | 2 | 3 | 4 | 5 | 6 | 7 | 8 | 9 | 10 | 11 | 12 | 13 | 14 | 15 | 16 | 17 | 18 | 19 | 20 | 21 | 1998 |  |
|  | 1999 |  |  |  |  |  |  | 0 | 1 | 2 | 3 | 4 | 5 | 6 | 7 | 8 | 9 | 10 | 11 | 12 | 13 | 14 | 15 | 16 | 17 | 18 | 19 | 20 | 1999 |  |
|  | 2000 |  |  |  |  |  |  |  | 0 | 1 | 2 | 3 | 4 | 5 | 6 | 7 | 8 | 9 | 10 | 11 | 12 | 13 | 14 | 15 | 16 | 17 | 18 | 19 | 2000 |  |
|  | 2001 |  |  |  |  |  |  |  |  | 0 | 1 | 2 | 3 | 4 | 5 | 6 | 7 | 8 | 9 | 10 | 11 | 12 | 13 | 14 | 15 | 16 | 17 | 18 | 2001 |  |
|  | 2002 |  |  |  |  |  |  |  |  |  | 0 | 1 | 2 | 3 | 4 | 5 | 6 | 7 | 8 | 9 | 10 | 11 | 12 | 13 | 14 | 15 | 16 | 17 | 2002 |  |
|  | 2003 |  |  |  |  |  |  |  |  |  |  | 0 | 1 | 2 | 3 | 4 | 5 | 6 | 7 | 8 | 9 | 10 | 11 | 12 | 13 | 14 | 15 | 16 | 2003 |  |
|  | 2004 |  |  |  |  |  |  |  |  |  |  |  | 0 | 1 | 2 | 3 | 4 | 5 | 6 | 7 | 8 | 9 | 10 | 11 | 12 | 13 | 14 | 15 | 2004 |  |
|  | 2005 |  |  |  |  |  |  |  |  |  |  |  |  | 0 | 1 | 2 | 3 | 4 | 5 | 6 | 7 | 8 | 9 | 10 | 11 | 12 | 13 | 14 | 2005 |  |
|  | 2006 |  |  |  |  |  |  |  |  |  |  |  |  |  | 0 | 1 | 2 | 3 | 4 | 5 | 6 | 7 | 8 | 9 | 10 | 11 | 12 | 13 | 2006 |  |
|  | 2007 |  |  |  |  |  |  |  |  |  |  |  |  |  |  | 0 | 1 | 2 | 3 | 4 | 5 | 6 | 7 | 8 | 9 | 10 | 11 | 12 | 2007 |  |
|  | 2008 |  |  |  |  |  |  |  |  |  |  |  |  |  |  |  | 0 | 1 | 2 | 3 | 4 | 5 | 6 | 7 | 8 | 9 | 10 | 11 | 2008 |  |
|  | 2009 |  |  |  |  |  |  |  |  |  |  |  |  |  |  |  |  | 0 | 1 | 2 | 3 | 4 | 5 | 6 | 7 | 8 | 9 | 10 | 2009 |  |
|  | 2010 |  |  |  |  |  |  |  |  |  |  |  |  |  |  |  |  |  | 0 | 1 | 2 | 3 | 4 | 5 | 6 | 7 | 8 | 9 | 2010 |  |
|  | 2011 |  |  |  |  |  |  |  |  |  |  |  |  |  |  |  |  |  |  | 0 | 1 | 2 | 3 | 4 | 5 | 6 | 7 | 8 | 2011 |  |
|  | 2012 |  |  |  |  |  |  |  |  |  |  |  |  |  |  |  |  |  |  |  | 0 | 1 | 2 | 3 | 4 | 5 | 6 | 7 | 2012 |  |
|  | 2013 |  |  |  |  |  |  |  |  |  |  |  |  |  |  |  |  |  |  |  |  | 0 | 1 | 2 | 3 | 4 | 5 | 6 | 2013 |  |
|  | 2014 |  |  |  |  |  |  |  |  |  |  |  |  |  |  |  |  |  |  |  |  |  | 0 | 1 | 2 | 3 | 4 | 5 | 2014 |  |
|  | 2015 |  |  |  |  |  |  |  |  |  |  |  |  |  |  |  |  |  |  |  |  |  |  | 0 | 1 | 2 | 3 | 4 | 2015 |  |
|  | 2016 |  |  |  |  |  |  |  |  |  |  |  |  |  |  |  |  |  |  |  |  |  |  |  | 0 | 1 | 2 | 3 | 2016 |  |
|  | 2017 |  |  |  |  |  |  |  |  |  |  |  |  |  |  |  |  |  |  |  |  |  |  |  |  | 0 | 1 | 2 | 2017 |  |
|  | 2018 |  |  |  |  |  |  |  |  |  |  |  |  |  |  |  |  |  |  |  |  |  |  |  |  |  | 0 | 1 | 2018 |  |

**Supplemental Figure 2**

|  |  | **Year of follow-up** | | | | | | | | | | | | | | | | | | | | | | | | | | |  |  |
| --- | --- | --- | --- | --- | --- | --- | --- | --- | --- | --- | --- | --- | --- | --- | --- | --- | --- | --- | --- | --- | --- | --- | --- | --- | --- | --- | --- | --- | --- | --- |
|  | **A** | 1993 | 1994 | 1995 | 1996 | 1997 | 1998 | 1999 | 2000 | 2001 | 2002 | 2003 | 2004 | 2005 | 2006 | 2007 | 2008 | 2009 | 2010 | 2011 | 2012 | 2013 | 2014 | 2015 | 2016 | 2017 | 2018 | 2019 |  |  |
| **Year of diagnosis** | 1993 |  |  |  |  |  |  |  |  |  |  |  |  |  |  |  |  |  |  |  |  |  |  |  |  |  |  |  | 1993 | **Year of diagnosis** |
|  | 1994 |  |  |  |  |  |  |  |  |  |  |  |  |  |  |  |  |  |  |  |  |  |  |  |  |  |  |  | 1994 |  |
|  | 1995 |  |  |  |  |  |  |  |  |  |  |  |  |  |  |  |  |  |  |  |  |  |  |  |  |  |  |  | 1995 |  |
|  | 1996 |  |  |  |  |  |  |  |  |  |  |  |  |  |  |  |  |  |  |  |  |  |  |  |  |  |  |  | 1996 |  |
|  | 1997 |  |  |  |  |  |  |  |  |  |  |  |  |  |  |  |  |  |  |  |  |  |  |  |  |  |  |  | 1997 |  |
|  | 1998 |  |  |  |  |  |  |  |  |  |  |  |  |  |  |  |  |  |  |  |  |  |  |  |  |  |  |  | 1998 |  |
|  | 1999 |  |  |  |  |  |  |  |  |  |  |  |  |  |  |  |  |  |  |  |  |  |  |  |  |  |  |  | 1999 |  |
|  | 2000 |  |  |  |  |  |  |  |  |  |  |  |  |  |  |  |  |  |  |  |  |  |  |  |  |  |  |  | 2000 |  |
|  | 2001 |  |  |  |  |  |  |  |  |  |  |  |  |  |  |  |  |  |  |  |  |  |  |  |  |  |  |  | 2001 |  |
|  | 2002 |  |  |  |  |  |  |  |  |  |  |  |  |  |  |  |  |  |  |  |  |  |  |  |  |  |  |  | 2002 |  |
|  | 2003 |  |  |  |  |  |  |  |  |  |  | 0 | 1 | 2 | 3 | 4 | 5 | 6 | 7 | 8 | 9 | 10 | 11 | 12 | 13 | 14 | 15 | 16 | 2003 |  |
|  | 2004 |  |  |  |  |  |  |  |  |  |  |  | 0 | 1 | 2 | 3 | 4 | 5 | 6 | 7 | 8 | 9 | 10 | 11 | 12 | 13 | 14 | 15 | 2004 |  |
|  | 2005 |  |  |  |  |  |  |  |  |  |  |  |  | 0 | 1 | 2 | 3 | 4 | 5 | 6 | 7 | 8 | 9 | 10 | 11 | 12 | 13 | 14 | 2005 |  |
|  | 2006 |  |  |  |  |  |  |  |  |  |  |  |  |  | 0 | 1 | 2 | 3 | 4 | 5 | 6 | 7 | 8 | 9 | 10 | 11 | 12 | 13 | 2006 |  |
|  | 2007 |  |  |  |  |  |  |  |  |  |  |  |  |  |  | 0 | 1 | 2 | 3 | 4 | 5 | 6 | 7 | 8 | 9 | 10 | 11 | 12 | 2007 |  |
|  | 2008 |  |  |  |  |  |  |  |  |  |  |  |  |  |  |  | 0 | 1 | 2 | 3 | 4 | 5 | 6 | 7 | 8 | 9 | 10 | 11 | 2008 |  |
|  | 2009 |  |  |  |  |  |  |  |  |  |  |  |  |  |  |  |  | 0 | 1 | 2 | 3 | 4 | 5 | 6 | 7 | 8 | 9 | 10 | 2009 |  |
|  | 2010 |  |  |  |  |  |  |  |  |  |  |  |  |  |  |  |  |  | 0 | 1 | 2 | 3 | 4 | 5 | 6 | 7 | 8 | 9 | 2010 |  |
|  | 2011 |  |  |  |  |  |  |  |  |  |  |  |  |  |  |  |  |  |  | 0 | 1 | 2 | 3 | 4 | 5 | 6 | 7 | 8 | 2011 |  |
|  | 2012 |  |  |  |  |  |  |  |  |  |  |  |  |  |  |  |  |  |  |  | 0 | 1 | 2 | 3 | 4 | 5 | 6 | 7 | 2012 |  |
|  | 2013 |  |  |  |  |  |  |  |  |  |  |  |  |  |  |  |  |  |  |  |  | 0 | 1 | 2 | 3 | 4 | 5 | 6 | 2013 |  |
|  | 2014 |  |  |  |  |  |  |  |  |  |  |  |  |  |  |  |  |  |  |  |  |  | 0 | 1 | 2 | 3 | 4 | 5 | 2014 |  |
|  | 2015 |  |  |  |  |  |  |  |  |  |  |  |  |  |  |  |  |  |  |  |  |  |  | 0 | 1 | 2 | 3 | 4 | 2015 |  |
|  | 2016 |  |  |  |  |  |  |  |  |  |  |  |  |  |  |  |  |  |  |  |  |  |  |  | 0 | 1 | 2 | 3 | 2016 |  |
|  | 2017 |  |  |  |  |  |  |  |  |  |  |  |  |  |  |  |  |  |  |  |  |  |  |  |  | 0 | 1 | 2 | 2017 |  |
|  | 2018 |  |  |  |  |  |  |  |  |  |  |  |  |  |  |  |  |  |  |  |  |  |  |  |  |  | 0 | 1 | 2018 |  |

**Supplemental Figure 2 – continued**

|  |  | **Year of follow-up** | | | | | | | | | | | | | | | | | | | | | | | | | | |  |  |
| --- | --- | --- | --- | --- | --- | --- | --- | --- | --- | --- | --- | --- | --- | --- | --- | --- | --- | --- | --- | --- | --- | --- | --- | --- | --- | --- | --- | --- | --- | --- |
|  | **B** | 1993 | 1994 | 1995 | 1996 | 1997 | 1998 | 1999 | 2000 | 2001 | 2002 | 2003 | 2004 | 2005 | 2006 | 2007 | 2008 | 2009 | 2010 | 2011 | 2012 | 2013 | 2014 | 2015 | 2016 | 2017 | 2018 | 2019 |  |  |
| **Year of diagnosis** | 1993 |  |  |  |  |  |  |  |  |  |  |  |  |  |  |  |  |  |  |  |  |  |  |  |  |  |  |  | 1993 | **Year of diagnosis** |
|  | 1994 |  |  |  |  |  |  |  |  |  |  |  |  |  |  |  |  |  |  |  |  |  |  |  |  |  |  |  | 1994 |  |
|  | 1995 |  |  |  |  |  |  |  |  |  |  |  |  |  |  |  |  |  |  |  |  |  |  |  |  |  |  |  | 1995 |  |
|  | 1996 |  |  |  |  |  |  |  |  |  |  |  |  |  |  |  |  |  |  |  |  |  |  |  |  |  |  |  | 1996 |  |
|  | 1997 |  |  |  |  |  |  |  |  |  |  |  |  |  |  |  |  |  |  |  |  |  |  |  |  |  |  |  | 1997 |  |
|  | 1998 |  |  |  |  |  |  |  |  |  |  |  |  |  |  |  |  |  |  |  |  |  |  |  |  |  |  |  | 1998 |  |
|  | 1999 |  |  |  |  |  |  |  |  |  |  |  |  |  |  |  |  |  |  |  |  |  |  |  |  |  |  |  | 1999 |  |
|  | 2000 |  |  |  |  |  |  |  |  |  |  |  |  |  |  |  |  |  |  |  |  |  |  |  |  |  |  |  | 2000 |  |
|  | 2001 |  |  |  |  |  |  |  |  |  |  |  |  |  |  |  |  |  |  |  |  |  |  |  |  |  |  |  | 2001 |  |
|  | 2002 |  |  |  |  |  |  |  |  |  |  | 0 | 1 | 2 | 3 | 4 | 5 | 6 | 7 | 8 | 9 | 10 | 11 | 12 | 13 | 14 | 15 | 16 | 2002 |  |
|  | 2003 |  |  |  |  |  |  |  |  |  |  |  | 0 | 1 | 2 | 3 | 4 | 5 | 6 | 7 | 8 | 9 | 10 | 11 | 12 | 13 | 14 | 15 | 2003 |  |
|  | 2004 |  |  |  |  |  |  |  |  |  |  |  |  | 0 | 1 | 2 | 3 | 4 | 5 | 6 | 7 | 8 | 9 | 10 | 11 | 12 | 13 | 14 | 2004 |  |
|  | 2005 |  |  |  |  |  |  |  |  |  |  |  |  |  | 0 | 1 | 2 | 3 | 4 | 5 | 6 | 7 | 8 | 9 | 10 | 11 | 12 | 13 | 2005 |  |
|  | 2006 |  |  |  |  |  |  |  |  |  |  |  |  |  |  | 0 | 1 | 2 | 3 | 4 | 5 | 6 | 7 | 8 | 9 | 10 | 11 | 12 | 2006 |  |
|  | 2007 |  |  |  |  |  |  |  |  |  |  |  |  |  |  |  | 0 | 1 | 2 | 3 | 4 | 5 | 6 | 7 | 8 | 9 | 10 | 11 | 2007 |  |
|  | 2008 |  |  |  |  |  |  |  |  |  |  |  |  |  |  |  |  | 0 | 1 | 2 | 3 | 4 | 5 | 6 | 7 | 8 | 9 | 10 | 2008 |  |
|  | 2009 |  |  |  |  |  |  |  |  |  |  |  |  |  |  |  |  |  | 0 | 1 | 2 | 3 | 4 | 5 | 6 | 7 | 8 | 9 | 2009 |  |
|  | 2010 |  |  |  |  |  |  |  |  |  |  |  |  |  |  |  |  |  |  | 0 | 1 | 2 | 3 | 4 | 5 | 6 | 7 | 8 | 2010 |  |
|  | 2011 |  |  |  |  |  |  |  |  |  |  |  |  |  |  |  |  |  |  |  | 0 | 1 | 2 | 3 | 4 | 5 | 6 | 7 | 2011 |  |
|  | 2012 |  |  |  |  |  |  |  |  |  |  |  |  |  |  |  |  |  |  |  |  | 0 | 1 | 2 | 3 | 4 | 5 | 6 | 2012 |  |
|  | 2013 |  |  |  |  |  |  |  |  |  |  |  |  |  |  |  |  |  |  |  |  |  | 0 | 1 | 2 | 3 | 4 | 5 | 2013 |  |
|  | 2014 |  |  |  |  |  |  |  |  |  |  |  |  |  |  |  |  |  |  |  |  |  |  | 0 | 1 | 2 | 3 | 4 | 2014 |  |
|  | 2015 |  |  |  |  |  |  |  |  |  |  |  |  |  |  |  |  |  |  |  |  |  |  |  | 0 | 1 | 2 | 3 | 2015 |  |
|  | 2016 |  |  |  |  |  |  |  |  |  |  |  |  |  |  |  |  |  |  |  |  |  |  |  |  | 0 | 1 | 2 | 2016 |  |
|  | 2017 |  |  |  |  |  |  |  |  |  |  |  |  |  |  |  |  |  |  |  |  |  |  |  |  |  | 0 | 1 | 2017 |  |
|  | 2018 |  |  |  |  |  |  |  |  |  |  |  |  |  |  |  |  |  |  |  |  |  |  |  |  |  |  | 0 | 2018 |  |

**Supplemental Figure 2 – continued**

|  |  | **Year of follow-up** | | | | | | | | | | | | | | | | | | | | | | | | | | |  |  |
| --- | --- | --- | --- | --- | --- | --- | --- | --- | --- | --- | --- | --- | --- | --- | --- | --- | --- | --- | --- | --- | --- | --- | --- | --- | --- | --- | --- | --- | --- | --- |
|  | **C** | 1993 | 1994 | 1995 | 1996 | 1997 | 1998 | 1999 | 2000 | 2001 | 2002 | 2003 | 2004 | 2005 | 2006 | 2007 | 2008 | 2009 | 2010 | 2011 | 2012 | 2013 | 2014 | 2015 | 2016 | 2017 | 2018 | 2019 |  |  |
| **Year of diagnosis** | 1993 |  |  |  |  |  |  |  |  |  |  |  |  |  |  |  |  |  |  |  |  |  |  |  |  |  |  |  | 1993 | **Year of diagnosis** |
|  | 1994 |  |  |  |  |  |  |  |  |  |  |  |  |  |  |  |  |  |  |  |  |  |  |  |  |  |  |  | 1994 |  |
|  | 1995 |  |  |  |  |  |  |  |  |  |  |  |  |  |  |  |  |  |  |  |  |  |  |  |  |  |  |  | 1995 |  |
|  | 1996 |  |  |  |  |  |  |  |  |  |  |  |  |  |  |  |  |  |  |  |  |  |  |  |  |  |  |  | 1996 |  |
|  | 1997 |  |  |  |  |  |  |  |  |  |  |  |  |  |  |  |  |  |  |  |  |  |  |  |  |  |  |  | 1997 |  |
|  | 1998 |  |  |  |  |  |  |  |  |  |  |  |  |  |  |  |  |  |  |  |  |  |  |  |  |  |  |  | 1998 |  |
|  | 1999 |  |  |  |  |  |  |  |  |  |  |  |  |  |  |  |  |  |  |  |  |  |  |  |  |  |  |  | 1999 |  |
|  | 2000 |  |  |  |  |  |  |  |  |  |  |  |  |  |  |  |  |  |  |  |  |  |  |  |  |  |  |  | 2000 |  |
|  | 2001 |  |  |  |  |  |  |  |  |  |  | 0 | 1 | 2 | 3 | 4 | 5 | 6 | 7 | 8 | 9 | 10 | 11 | 12 | 13 | 14 | 15 | 16 | 2001 |  |
|  | 2002 |  |  |  |  |  |  |  |  |  |  |  | 0 | 1 | 2 | 3 | 4 | 5 | 6 | 7 | 8 | 9 | 10 | 11 | 12 | 13 | 14 | 15 | 2002 |  |
|  | 2003 |  |  |  |  |  |  |  |  |  |  |  |  | 0 | 1 | 2 | 3 | 4 | 5 | 6 | 7 | 8 | 9 | 10 | 11 | 12 | 13 | 14 | 2003 |  |
|  | 2004 |  |  |  |  |  |  |  |  |  |  |  |  |  | 0 | 1 | 2 | 3 | 4 | 5 | 6 | 7 | 8 | 9 | 10 | 11 | 12 | 13 | 2004 |  |
|  | 2005 |  |  |  |  |  |  |  |  |  |  |  |  |  |  | 0 | 1 | 2 | 3 | 4 | 5 | 6 | 7 | 8 | 9 | 10 | 11 | 12 | 2005 |  |
|  | 2006 |  |  |  |  |  |  |  |  |  |  |  |  |  |  |  | 0 | 1 | 2 | 3 | 4 | 5 | 6 | 7 | 8 | 9 | 10 | 11 | 2006 |  |
|  | 2007 |  |  |  |  |  |  |  |  |  |  |  |  |  |  |  |  | 0 | 1 | 2 | 3 | 4 | 5 | 6 | 7 | 8 | 9 | 10 | 2007 |  |
|  | 2008 |  |  |  |  |  |  |  |  |  |  |  |  |  |  |  |  |  | 0 | 1 | 2 | 3 | 4 | 5 | 6 | 7 | 8 | 9 | 2008 |  |
|  | 2009 |  |  |  |  |  |  |  |  |  |  |  |  |  |  |  |  |  |  | 0 | 1 | 2 | 3 | 4 | 5 | 6 | 7 | 8 | 2009 |  |
|  | 2010 |  |  |  |  |  |  |  |  |  |  |  |  |  |  |  |  |  |  |  | 0 | 1 | 2 | 3 | 4 | 5 | 6 | 7 | 2010 |  |
|  | 2011 |  |  |  |  |  |  |  |  |  |  |  |  |  |  |  |  |  |  |  |  | 0 | 1 | 2 | 3 | 4 | 5 | 6 | 2011 |  |
|  | 2012 |  |  |  |  |  |  |  |  |  |  |  |  |  |  |  |  |  |  |  |  |  | 0 | 1 | 2 | 3 | 4 | 5 | 2012 |  |
|  | 2013 |  |  |  |  |  |  |  |  |  |  |  |  |  |  |  |  |  |  |  |  |  |  | 0 | 1 | 2 | 3 | 4 | 2013 |  |
|  | 2014 |  |  |  |  |  |  |  |  |  |  |  |  |  |  |  |  |  |  |  |  |  |  |  | 0 | 1 | 2 | 3 | 2014 |  |
|  | 2015 |  |  |  |  |  |  |  |  |  |  |  |  |  |  |  |  |  |  |  |  |  |  |  |  | 0 | 1 | 2 | 2015 |  |
|  | 2016 |  |  |  |  |  |  |  |  |  |  |  |  |  |  |  |  |  |  |  |  |  |  |  |  |  | 0 | 1 | 2016 |  |
|  | 2017 |  |  |  |  |  |  |  |  |  |  |  |  |  |  |  |  |  |  |  |  |  |  |  |  |  |  | 0 | 2017 |  |
|  | 2018 |  |  |  |  |  |  |  |  |  |  |  |  |  |  |  |  |  |  |  |  |  |  |  |  |  |  |  | 2018 |  |

**Supplemental Figure 2 – continued**

|  |  | **Year of follow-up** | | | | | | | | | | | | | | | | | | | | | | | | | | |  |  |
| --- | --- | --- | --- | --- | --- | --- | --- | --- | --- | --- | --- | --- | --- | --- | --- | --- | --- | --- | --- | --- | --- | --- | --- | --- | --- | --- | --- | --- | --- | --- |
|  | **D** | 1993 | 1994 | 1995 | 1996 | 1997 | 1998 | 1999 | 2000 | 2001 | 2002 | 2003 | 2004 | 2005 | 2006 | 2007 | 2008 | 2009 | 2010 | 2011 | 2012 | 2013 | 2014 | 2015 | 2016 | 2017 | 2018 | 2019 |  |  |
| **Year of diagnosis** | 1993 |  |  |  |  |  |  |  |  |  |  |  |  |  |  |  |  |  |  |  |  |  |  |  |  |  |  |  | 1993 | **Year of diagnosis** |
|  | 1994 |  |  |  |  |  |  |  |  |  |  |  |  |  |  |  |  |  |  |  |  |  |  |  |  |  |  |  | 1994 |  |
|  | 1995 |  |  |  |  |  |  |  |  |  |  |  |  |  |  |  |  |  |  |  |  |  |  |  |  |  |  |  | 1995 |  |
|  | 1996 |  |  |  |  |  |  |  |  |  |  |  |  |  |  |  |  |  |  |  |  |  |  |  |  |  |  |  | 1996 |  |
|  | 1997 |  |  |  |  |  |  |  |  |  |  |  |  |  |  |  |  |  |  |  |  |  |  |  |  |  |  |  | 1997 |  |
|  | 1998 |  |  |  |  |  |  |  |  |  |  |  |  |  |  |  |  |  |  |  |  |  |  |  |  |  |  |  | 1998 |  |
|  | 1999 |  |  |  |  |  |  |  |  |  |  |  |  |  |  |  |  |  |  |  |  |  |  |  |  |  |  |  | 1999 |  |
|  | 2000 |  |  |  |  |  |  |  |  |  |  | 0 | 1 | 2 | 3 | 4 | 5 | 6 | 7 | 8 | 9 | 10 | 11 | 12 | 13 | 14 | 15 | 16 | 2000 |  |
|  | 2001 |  |  |  |  |  |  |  |  |  |  |  | 0 | 1 | 2 | 3 | 4 | 5 | 6 | 7 | 8 | 9 | 10 | 11 | 12 | 13 | 14 | 15 | 2001 |  |
|  | 2002 |  |  |  |  |  |  |  |  |  |  |  |  | 0 | 1 | 2 | 3 | 4 | 5 | 6 | 7 | 8 | 9 | 10 | 11 | 12 | 13 | 14 | 2002 |  |
|  | 2003 |  |  |  |  |  |  |  |  |  |  |  |  |  | 0 | 1 | 2 | 3 | 4 | 5 | 6 | 7 | 8 | 9 | 10 | 11 | 12 | 13 | 2003 |  |
|  | 2004 |  |  |  |  |  |  |  |  |  |  |  |  |  |  | 0 | 1 | 2 | 3 | 4 | 5 | 6 | 7 | 8 | 9 | 10 | 11 | 12 | 2004 |  |
|  | 2005 |  |  |  |  |  |  |  |  |  |  |  |  |  |  |  | 0 | 1 | 2 | 3 | 4 | 5 | 6 | 7 | 8 | 9 | 10 | 11 | 2005 |  |
|  | 2006 |  |  |  |  |  |  |  |  |  |  |  |  |  |  |  |  | 0 | 1 | 2 | 3 | 4 | 5 | 6 | 7 | 8 | 9 | 10 | 2006 |  |
|  | 2007 |  |  |  |  |  |  |  |  |  |  |  |  |  |  |  |  |  | 0 | 1 | 2 | 3 | 4 | 5 | 6 | 7 | 8 | 9 | 2007 |  |
|  | 2008 |  |  |  |  |  |  |  |  |  |  |  |  |  |  |  |  |  |  | 0 | 1 | 2 | 3 | 4 | 5 | 6 | 7 | 8 | 2008 |  |
|  | 2009 |  |  |  |  |  |  |  |  |  |  |  |  |  |  |  |  |  |  |  | 0 | 1 | 2 | 3 | 4 | 5 | 6 | 7 | 2009 |  |
|  | 2010 |  |  |  |  |  |  |  |  |  |  |  |  |  |  |  |  |  |  |  |  | 0 | 1 | 2 | 3 | 4 | 5 | 6 | 2010 |  |
|  | 2011 |  |  |  |  |  |  |  |  |  |  |  |  |  |  |  |  |  |  |  |  |  | 0 | 1 | 2 | 3 | 4 | 5 | 2011 |  |
|  | 2012 |  |  |  |  |  |  |  |  |  |  |  |  |  |  |  |  |  |  |  |  |  |  | 0 | 1 | 2 | 3 | 4 | 2012 |  |
|  | 2013 |  |  |  |  |  |  |  |  |  |  |  |  |  |  |  |  |  |  |  |  |  |  |  | 0 | 1 | 2 | 3 | 2013 |  |
|  | 2014 |  |  |  |  |  |  |  |  |  |  |  |  |  |  |  |  |  |  |  |  |  |  |  |  | 0 | 1 | 2 | 2014 |  |
|  | 2015 |  |  |  |  |  |  |  |  |  |  |  |  |  |  |  |  |  |  |  |  |  |  |  |  |  | 0 | 1 | 2015 |  |
|  | 2016 |  |  |  |  |  |  |  |  |  |  |  |  |  |  |  |  |  |  |  |  |  |  |  |  |  |  | 0 | 2016 |  |
|  | 2017 |  |  |  |  |  |  |  |  |  |  |  |  |  |  |  |  |  |  |  |  |  |  |  |  |  |  |  | 2017 |  |
|  | 2018 |  |  |  |  |  |  |  |  |  |  |  |  |  |  |  |  |  |  |  |  |  |  |  |  |  |  |  | 2018 |  |

**Supplemental Figure 2 – continued**

|  |  | **Year of follow-up** | | | | | | | | | | | | | | | | | | | | | | | | | | |  |  |
| --- | --- | --- | --- | --- | --- | --- | --- | --- | --- | --- | --- | --- | --- | --- | --- | --- | --- | --- | --- | --- | --- | --- | --- | --- | --- | --- | --- | --- | --- | --- |
|  | **E** | 1993 | 1994 | 1995 | 1996 | 1997 | 1998 | 1999 | 2000 | 2001 | 2002 | 2003 | 2004 | 2005 | 2006 | 2007 | 2008 | 2009 | 2010 | 2011 | 2012 | 2013 | 2014 | 2015 | 2016 | 2017 | 2018 | 2019 |  |  |
| **Year of diagnosis** | 1993 |  |  |  |  |  |  |  |  |  |  |  |  |  |  |  |  |  |  |  |  |  |  |  |  |  |  |  | 1993 | **Year of diagnosis** |
|  | 1994 |  |  |  |  |  |  |  |  |  |  |  |  |  |  |  |  |  |  |  |  |  |  |  |  |  |  |  | 1994 |  |
|  | 1995 |  |  |  |  |  |  |  |  |  |  |  |  |  |  |  |  |  |  |  |  |  |  |  |  |  |  |  | 1995 |  |
|  | 1996 |  |  |  |  |  |  |  |  |  |  |  |  |  |  |  |  |  |  |  |  |  |  |  |  |  |  |  | 1996 |  |
|  | 1997 |  |  |  |  |  |  |  |  |  |  |  |  |  |  |  |  |  |  |  |  |  |  |  |  |  |  |  | 1997 |  |
|  | 1998 |  |  |  |  |  |  |  |  |  |  |  |  |  |  |  |  |  |  |  |  |  |  |  |  |  |  |  | 1998 |  |
|  | 1999 |  |  |  |  |  |  |  |  |  |  | 0 | 1 | 2 | 3 | 4 | 5 | 6 | 7 | 8 | 9 | 10 | 11 | 12 | 13 | 14 | 15 | 16 | 1999 |  |
|  | 2000 |  |  |  |  |  |  |  |  |  |  |  | 0 | 1 | 2 | 3 | 4 | 5 | 6 | 7 | 8 | 9 | 10 | 11 | 12 | 13 | 14 | 15 | 2000 |  |
|  | 2001 |  |  |  |  |  |  |  |  |  |  |  |  | 0 | 1 | 2 | 3 | 4 | 5 | 6 | 7 | 8 | 9 | 10 | 11 | 12 | 13 | 14 | 2001 |  |
|  | 2002 |  |  |  |  |  |  |  |  |  |  |  |  |  | 0 | 1 | 2 | 3 | 4 | 5 | 6 | 7 | 8 | 9 | 10 | 11 | 12 | 13 | 2002 |  |
|  | 2003 |  |  |  |  |  |  |  |  |  |  |  |  |  |  | 0 | 1 | 2 | 3 | 4 | 5 | 6 | 7 | 8 | 9 | 10 | 11 | 12 | 2003 |  |
|  | 2004 |  |  |  |  |  |  |  |  |  |  |  |  |  |  |  | 0 | 1 | 2 | 3 | 4 | 5 | 6 | 7 | 8 | 9 | 10 | 11 | 2004 |  |
|  | 2005 |  |  |  |  |  |  |  |  |  |  |  |  |  |  |  |  | 0 | 1 | 2 | 3 | 4 | 5 | 6 | 7 | 8 | 9 | 10 | 2005 |  |
|  | 2006 |  |  |  |  |  |  |  |  |  |  |  |  |  |  |  |  |  | 0 | 1 | 2 | 3 | 4 | 5 | 6 | 7 | 8 | 9 | 2006 |  |
|  | 2007 |  |  |  |  |  |  |  |  |  |  |  |  |  |  |  |  |  |  | 0 | 1 | 2 | 3 | 4 | 5 | 6 | 7 | 8 | 2007 |  |
|  | 2008 |  |  |  |  |  |  |  |  |  |  |  |  |  |  |  |  |  |  |  | 0 | 1 | 2 | 3 | 4 | 5 | 6 | 7 | 2008 |  |
|  | 2009 |  |  |  |  |  |  |  |  |  |  |  |  |  |  |  |  |  |  |  |  | 0 | 1 | 2 | 3 | 4 | 5 | 6 | 2009 |  |
|  | 2010 |  |  |  |  |  |  |  |  |  |  |  |  |  |  |  |  |  |  |  |  |  | 0 | 1 | 2 | 3 | 4 | 5 | 2010 |  |
|  | 2011 |  |  |  |  |  |  |  |  |  |  |  |  |  |  |  |  |  |  |  |  |  |  | 0 | 1 | 2 | 3 | 4 | 2011 |  |
|  | 2012 |  |  |  |  |  |  |  |  |  |  |  |  |  |  |  |  |  |  |  |  |  |  |  | 0 | 1 | 2 | 3 | 2012 |  |
|  | 2013 |  |  |  |  |  |  |  |  |  |  |  |  |  |  |  |  |  |  |  |  |  |  |  |  | 0 | 1 | 2 | 2013 |  |
|  | 2014 |  |  |  |  |  |  |  |  |  |  |  |  |  |  |  |  |  |  |  |  |  |  |  |  |  | 0 | 1 | 2014 |  |
|  | 2015 |  |  |  |  |  |  |  |  |  |  |  |  |  |  |  |  |  |  |  |  |  |  |  |  |  |  | 0 | 2015 |  |
|  | 2016 |  |  |  |  |  |  |  |  |  |  |  |  |  |  |  |  |  |  |  |  |  |  |  |  |  |  |  | 2016 |  |
|  | 2017 |  |  |  |  |  |  |  |  |  |  |  |  |  |  |  |  |  |  |  |  |  |  |  |  |  |  |  | 2017 |  |
|  | 2018 |  |  |  |  |  |  |  |  |  |  |  |  |  |  |  |  |  |  |  |  |  |  |  |  |  |  |  | 2018 |  |

**Supplemental Figure 2 – continued**

|  |  | **Year of follow-up** | | | | | | | | | | | | | | | | | | | | | | | | | | |  |  |
| --- | --- | --- | --- | --- | --- | --- | --- | --- | --- | --- | --- | --- | --- | --- | --- | --- | --- | --- | --- | --- | --- | --- | --- | --- | --- | --- | --- | --- | --- | --- |
|  | **F** | 1993 | 1994 | 1995 | 1996 | 1997 | 1998 | 1999 | 2000 | 2001 | 2002 | 2003 | 2004 | 2005 | 2006 | 2007 | 2008 | 2009 | 2010 | 2011 | 2012 | 2013 | 2014 | 2015 | 2016 | 2017 | 2018 | 2019 |  |  |
| **Year of diagnosis** | 1993 |  |  |  |  |  |  |  |  |  |  |  |  |  |  |  |  |  |  |  |  |  |  |  |  |  |  |  | 1993 | **Year of diagnosis** |
|  | 1994 |  |  |  |  |  |  |  |  |  |  |  |  |  |  |  |  |  |  |  |  |  |  |  |  |  |  |  | 1994 |  |
|  | 1995 |  |  |  |  |  |  |  |  |  |  |  |  |  |  |  |  |  |  |  |  |  |  |  |  |  |  |  | 1995 |  |
|  | 1996 |  |  |  |  |  |  |  |  |  |  |  |  |  |  |  |  |  |  |  |  |  |  |  |  |  |  |  | 1996 |  |
|  | 1997 |  |  |  |  |  |  |  |  |  |  |  |  |  |  |  |  |  |  |  |  |  |  |  |  |  |  |  | 1997 |  |
|  | 1998 |  |  |  |  |  |  |  |  |  |  | 0 | 1 | 2 | 3 | 4 | 5 | 6 | 7 | 8 | 9 | 10 | 11 | 12 | 13 | 14 | 15 | 16 | 1998 |  |
|  | 1999 |  |  |  |  |  |  |  |  |  |  |  | 0 | 1 | 2 | 3 | 4 | 5 | 6 | 7 | 8 | 9 | 10 | 11 | 12 | 13 | 14 | 15 | 1999 |  |
|  | 2000 |  |  |  |  |  |  |  |  |  |  |  |  | 0 | 1 | 2 | 3 | 4 | 5 | 6 | 7 | 8 | 9 | 10 | 11 | 12 | 13 | 14 | 2000 |  |
|  | 2001 |  |  |  |  |  |  |  |  |  |  |  |  |  | 0 | 1 | 2 | 3 | 4 | 5 | 6 | 7 | 8 | 9 | 10 | 11 | 12 | 13 | 2001 |  |
|  | 2002 |  |  |  |  |  |  |  |  |  |  |  |  |  |  | 0 | 1 | 2 | 3 | 4 | 5 | 6 | 7 | 8 | 9 | 10 | 11 | 12 | 2002 |  |
|  | 2003 |  |  |  |  |  |  |  |  |  |  |  |  |  |  |  | 0 | 1 | 2 | 3 | 4 | 5 | 6 | 7 | 8 | 9 | 10 | 11 | 2003 |  |
|  | 2004 |  |  |  |  |  |  |  |  |  |  |  |  |  |  |  |  | 0 | 1 | 2 | 3 | 4 | 5 | 6 | 7 | 8 | 9 | 10 | 2004 |  |
|  | 2005 |  |  |  |  |  |  |  |  |  |  |  |  |  |  |  |  |  | 0 | 1 | 2 | 3 | 4 | 5 | 6 | 7 | 8 | 9 | 2005 |  |
|  | 2006 |  |  |  |  |  |  |  |  |  |  |  |  |  |  |  |  |  |  | 0 | 1 | 2 | 3 | 4 | 5 | 6 | 7 | 8 | 2006 |  |
|  | 2007 |  |  |  |  |  |  |  |  |  |  |  |  |  |  |  |  |  |  |  | 0 | 1 | 2 | 3 | 4 | 5 | 6 | 7 | 2007 |  |
|  | 2008 |  |  |  |  |  |  |  |  |  |  |  |  |  |  |  |  |  |  |  |  | 0 | 1 | 2 | 3 | 4 | 5 | 6 | 2008 |  |
|  | 2009 |  |  |  |  |  |  |  |  |  |  |  |  |  |  |  |  |  |  |  |  |  | 0 | 1 | 2 | 3 | 4 | 5 | 2009 |  |
|  | 2010 |  |  |  |  |  |  |  |  |  |  |  |  |  |  |  |  |  |  |  |  |  |  | 0 | 1 | 2 | 3 | 4 | 2010 |  |
|  | 2011 |  |  |  |  |  |  |  |  |  |  |  |  |  |  |  |  |  |  |  |  |  |  |  | 0 | 1 | 2 | 3 | 2011 |  |
|  | 2012 |  |  |  |  |  |  |  |  |  |  |  |  |  |  |  |  |  |  |  |  |  |  |  |  | 0 | 1 | 2 | 2012 |  |
|  | 2013 |  |  |  |  |  |  |  |  |  |  |  |  |  |  |  |  |  |  |  |  |  |  |  |  |  | 0 | 1 | 2013 |  |
|  | 2014 |  |  |  |  |  |  |  |  |  |  |  |  |  |  |  |  |  |  |  |  |  |  |  |  |  |  | 0 | 2014 |  |
|  | 2015 |  |  |  |  |  |  |  |  |  |  |  |  |  |  |  |  |  |  |  |  |  |  |  |  |  |  |  | 2015 |  |
|  | 2016 |  |  |  |  |  |  |  |  |  |  |  |  |  |  |  |  |  |  |  |  |  |  |  |  |  |  |  | 2016 |  |
|  | 2017 |  |  |  |  |  |  |  |  |  |  |  |  |  |  |  |  |  |  |  |  |  |  |  |  |  |  |  | 2017 |  |
|  | 2018 |  |  |  |  |  |  |  |  |  |  |  |  |  |  |  |  |  |  |  |  |  |  |  |  |  |  |  | 2018 |  |

**Supplemental Figure 2 – continued**

|  |  | **Year of follow-up** | | | | | | | | | | | | | | | | | | | | | | | | | | |  |  |
| --- | --- | --- | --- | --- | --- | --- | --- | --- | --- | --- | --- | --- | --- | --- | --- | --- | --- | --- | --- | --- | --- | --- | --- | --- | --- | --- | --- | --- | --- | --- |
|  | **G** | 1993 | 1994 | 1995 | 1996 | 1997 | 1998 | 1999 | 2000 | 2001 | 2002 | 2003 | 2004 | 2005 | 2006 | 2007 | 2008 | 2009 | 2010 | 2011 | 2012 | 2013 | 2014 | 2015 | 2016 | 2017 | 2018 | 2019 |  |  |
| **Year of diagnosis** | 1993 |  |  |  |  |  |  |  |  |  |  |  |  |  |  |  |  |  |  |  |  |  |  |  |  |  |  |  | 1993 | **Year of diagnosis** |
|  | 1994 |  |  |  |  |  |  |  |  |  |  |  |  |  |  |  |  |  |  |  |  |  |  |  |  |  |  |  | 1994 |  |
|  | 1995 |  |  |  |  |  |  |  |  |  |  |  |  |  |  |  |  |  |  |  |  |  |  |  |  |  |  |  | 1995 |  |
|  | 1996 |  |  |  |  |  |  |  |  |  |  |  |  |  |  |  |  |  |  |  |  |  |  |  |  |  |  |  | 1996 |  |
|  | 1997 |  |  |  |  |  |  |  |  |  |  | 0 | 1 | 2 | 3 | 4 | 5 | 6 | 7 | 8 | 9 | 10 | 11 | 12 | 13 | 14 | 15 | 16 | 1997 |  |
|  | 1998 |  |  |  |  |  |  |  |  |  |  |  | 0 | 1 | 2 | 3 | 4 | 5 | 6 | 7 | 8 | 9 | 10 | 11 | 12 | 13 | 14 | 15 | 1998 |  |
|  | 1999 |  |  |  |  |  |  |  |  |  |  |  |  | 0 | 1 | 2 | 3 | 4 | 5 | 6 | 7 | 8 | 9 | 10 | 11 | 12 | 13 | 14 | 1999 |  |
|  | 2000 |  |  |  |  |  |  |  |  |  |  |  |  |  | 0 | 1 | 2 | 3 | 4 | 5 | 6 | 7 | 8 | 9 | 10 | 11 | 12 | 13 | 2000 |  |
|  | 2001 |  |  |  |  |  |  |  |  |  |  |  |  |  |  | 0 | 1 | 2 | 3 | 4 | 5 | 6 | 7 | 8 | 9 | 10 | 11 | 12 | 2001 |  |
|  | 2002 |  |  |  |  |  |  |  |  |  |  |  |  |  |  |  | 0 | 1 | 2 | 3 | 4 | 5 | 6 | 7 | 8 | 9 | 10 | 11 | 2002 |  |
|  | 2003 |  |  |  |  |  |  |  |  |  |  |  |  |  |  |  |  | 0 | 1 | 2 | 3 | 4 | 5 | 6 | 7 | 8 | 9 | 10 | 2003 |  |
|  | 2004 |  |  |  |  |  |  |  |  |  |  |  |  |  |  |  |  |  | 0 | 1 | 2 | 3 | 4 | 5 | 6 | 7 | 8 | 9 | 2004 |  |
|  | 2005 |  |  |  |  |  |  |  |  |  |  |  |  |  |  |  |  |  |  | 0 | 1 | 2 | 3 | 4 | 5 | 6 | 7 | 8 | 2005 |  |
|  | 2006 |  |  |  |  |  |  |  |  |  |  |  |  |  |  |  |  |  |  |  | 0 | 1 | 2 | 3 | 4 | 5 | 6 | 7 | 2006 |  |
|  | 2007 |  |  |  |  |  |  |  |  |  |  |  |  |  |  |  |  |  |  |  |  | 0 | 1 | 2 | 3 | 4 | 5 | 6 | 2007 |  |
|  | 2008 |  |  |  |  |  |  |  |  |  |  |  |  |  |  |  |  |  |  |  |  |  | 0 | 1 | 2 | 3 | 4 | 5 | 2008 |  |
|  | 2009 |  |  |  |  |  |  |  |  |  |  |  |  |  |  |  |  |  |  |  |  |  |  | 0 | 1 | 2 | 3 | 4 | 2009 |  |
|  | 2010 |  |  |  |  |  |  |  |  |  |  |  |  |  |  |  |  |  |  |  |  |  |  |  | 0 | 1 | 2 | 3 | 2010 |  |
|  | 2011 |  |  |  |  |  |  |  |  |  |  |  |  |  |  |  |  |  |  |  |  |  |  |  |  | 0 | 1 | 2 | 2011 |  |
|  | 2012 |  |  |  |  |  |  |  |  |  |  |  |  |  |  |  |  |  |  |  |  |  |  |  |  |  | 0 | 1 | 2012 |  |
|  | 2013 |  |  |  |  |  |  |  |  |  |  |  |  |  |  |  |  |  |  |  |  |  |  |  |  |  |  | 0 | 2013 |  |
|  | 2014 |  |  |  |  |  |  |  |  |  |  |  |  |  |  |  |  |  |  |  |  |  |  |  |  |  |  |  | 2014 |  |
|  | 2015 |  |  |  |  |  |  |  |  |  |  |  |  |  |  |  |  |  |  |  |  |  |  |  |  |  |  |  | 2015 |  |
|  | 2016 |  |  |  |  |  |  |  |  |  |  |  |  |  |  |  |  |  |  |  |  |  |  |  |  |  |  |  | 2016 |  |
|  | 2017 |  |  |  |  |  |  |  |  |  |  |  |  |  |  |  |  |  |  |  |  |  |  |  |  |  |  |  | 2017 |  |
|  | 2018 |  |  |  |  |  |  |  |  |  |  |  |  |  |  |  |  |  |  |  |  |  |  |  |  |  |  |  | 2018 |  |

**Supplemental Figure 2 – continued**

|  |  | **Year of follow-up** | | | | | | | | | | | | | | | | | | | | | | | | | | |  |  |
| --- | --- | --- | --- | --- | --- | --- | --- | --- | --- | --- | --- | --- | --- | --- | --- | --- | --- | --- | --- | --- | --- | --- | --- | --- | --- | --- | --- | --- | --- | --- |
|  | **H** | 1993 | 1994 | 1995 | 1996 | 1997 | 1998 | 1999 | 2000 | 2001 | 2002 | 2003 | 2004 | 2005 | 2006 | 2007 | 2008 | 2009 | 2010 | 2011 | 2012 | 2013 | 2014 | 2015 | 2016 | 2017 | 2018 | 2019 |  |  |
| **Year of diagnosis** | 1993 |  |  |  |  |  |  |  |  |  |  |  |  |  |  |  |  |  |  |  |  |  |  |  |  |  |  |  | 1993 | **Year of diagnosis** |
|  | 1994 |  |  |  |  |  |  |  |  |  |  |  |  |  |  |  |  |  |  |  |  |  |  |  |  |  |  |  | 1994 |  |
|  | 1995 |  |  |  |  |  |  |  |  |  |  |  |  |  |  |  |  |  |  |  |  |  |  |  |  |  |  |  | 1995 |  |
|  | 1996 |  |  |  |  |  |  |  |  |  |  | 0 | 1 | 2 | 3 | 4 | 5 | 6 | 7 | 8 | 9 | 10 | 11 | 12 | 13 | 14 | 15 | 16 | 1996 |  |
|  | 1997 |  |  |  |  |  |  |  |  |  |  |  | 0 | 1 | 2 | 3 | 4 | 5 | 6 | 7 | 8 | 9 | 10 | 11 | 12 | 13 | 14 | 15 | 1997 |  |
|  | 1998 |  |  |  |  |  |  |  |  |  |  |  |  | 0 | 1 | 2 | 3 | 4 | 5 | 6 | 7 | 8 | 9 | 10 | 11 | 12 | 13 | 14 | 1998 |  |
|  | 1999 |  |  |  |  |  |  |  |  |  |  |  |  |  | 0 | 1 | 2 | 3 | 4 | 5 | 6 | 7 | 8 | 9 | 10 | 11 | 12 | 13 | 1999 |  |
|  | 2000 |  |  |  |  |  |  |  |  |  |  |  |  |  |  | 0 | 1 | 2 | 3 | 4 | 5 | 6 | 7 | 8 | 9 | 10 | 11 | 12 | 2000 |  |
|  | 2001 |  |  |  |  |  |  |  |  |  |  |  |  |  |  |  | 0 | 1 | 2 | 3 | 4 | 5 | 6 | 7 | 8 | 9 | 10 | 11 | 2001 |  |
|  | 2002 |  |  |  |  |  |  |  |  |  |  |  |  |  |  |  |  | 0 | 1 | 2 | 3 | 4 | 5 | 6 | 7 | 8 | 9 | 10 | 2002 |  |
|  | 2003 |  |  |  |  |  |  |  |  |  |  |  |  |  |  |  |  |  | 0 | 1 | 2 | 3 | 4 | 5 | 6 | 7 | 8 | 9 | 2003 |  |
|  | 2004 |  |  |  |  |  |  |  |  |  |  |  |  |  |  |  |  |  |  | 0 | 1 | 2 | 3 | 4 | 5 | 6 | 7 | 8 | 2004 |  |
|  | 2005 |  |  |  |  |  |  |  |  |  |  |  |  |  |  |  |  |  |  |  | 0 | 1 | 2 | 3 | 4 | 5 | 6 | 7 | 2005 |  |
|  | 2006 |  |  |  |  |  |  |  |  |  |  |  |  |  |  |  |  |  |  |  |  | 0 | 1 | 2 | 3 | 4 | 5 | 6 | 2006 |  |
|  | 2007 |  |  |  |  |  |  |  |  |  |  |  |  |  |  |  |  |  |  |  |  |  | 0 | 1 | 2 | 3 | 4 | 5 | 2007 |  |
|  | 2008 |  |  |  |  |  |  |  |  |  |  |  |  |  |  |  |  |  |  |  |  |  |  | 0 | 1 | 2 | 3 | 4 | 2008 |  |
|  | 2009 |  |  |  |  |  |  |  |  |  |  |  |  |  |  |  |  |  |  |  |  |  |  |  | 0 | 1 | 2 | 3 | 2009 |  |
|  | 2010 |  |  |  |  |  |  |  |  |  |  |  |  |  |  |  |  |  |  |  |  |  |  |  |  | 0 | 1 | 2 | 2010 |  |
|  | 2011 |  |  |  |  |  |  |  |  |  |  |  |  |  |  |  |  |  |  |  |  |  |  |  |  |  | 0 | 1 | 2011 |  |
|  | 2012 |  |  |  |  |  |  |  |  |  |  |  |  |  |  |  |  |  |  |  |  |  |  |  |  |  |  | 0 | 2012 |  |
|  | 2013 |  |  |  |  |  |  |  |  |  |  |  |  |  |  |  |  |  |  |  |  |  |  |  |  |  |  |  | 2013 |  |
|  | 2014 |  |  |  |  |  |  |  |  |  |  |  |  |  |  |  |  |  |  |  |  |  |  |  |  |  |  |  | 2014 |  |
|  | 2015 |  |  |  |  |  |  |  |  |  |  |  |  |  |  |  |  |  |  |  |  |  |  |  |  |  |  |  | 2015 |  |
|  | 2016 |  |  |  |  |  |  |  |  |  |  |  |  |  |  |  |  |  |  |  |  |  |  |  |  |  |  |  | 2016 |  |
|  | 2017 |  |  |  |  |  |  |  |  |  |  |  |  |  |  |  |  |  |  |  |  |  |  |  |  |  |  |  | 2017 |  |
|  | 2018 |  |  |  |  |  |  |  |  |  |  |  |  |  |  |  |  |  |  |  |  |  |  |  |  |  |  |  | 2018 |  |

**Supplemental Figure 2 – continued**

|  |  | **Year of follow-up** | | | | | | | | | | | | | | | | | | | | | | | | | | |  |  |
| --- | --- | --- | --- | --- | --- | --- | --- | --- | --- | --- | --- | --- | --- | --- | --- | --- | --- | --- | --- | --- | --- | --- | --- | --- | --- | --- | --- | --- | --- | --- |
|  | **I** | 1993 | 1994 | 1995 | 1996 | 1997 | 1998 | 1999 | 2000 | 2001 | 2002 | 2003 | 2004 | 2005 | 2006 | 2007 | 2008 | 2009 | 2010 | 2011 | 2012 | 2013 | 2014 | 2015 | 2016 | 2017 | 2018 | 2019 |  |  |
| **Year of diagnosis** | 1993 |  |  |  |  |  |  |  |  |  |  |  |  |  |  |  |  |  |  |  |  |  |  |  |  |  |  |  | 1993 | **Year of diagnosis** |
|  | 1994 |  |  |  |  |  |  |  |  |  |  |  |  |  |  |  |  |  |  |  |  |  |  |  |  |  |  |  | 1994 |  |
|  | 1995 |  |  |  |  |  |  |  |  |  |  | 0 | 1 | 2 | 3 | 4 | 5 | 6 | 7 | 8 | 9 | 10 | 11 | 12 | 13 | 14 | 15 | 16 | 1995 |  |
|  | 1996 |  |  |  |  |  |  |  |  |  |  |  | 0 | 1 | 2 | 3 | 4 | 5 | 6 | 7 | 8 | 9 | 10 | 11 | 12 | 13 | 14 | 15 | 1996 |  |
|  | 1997 |  |  |  |  |  |  |  |  |  |  |  |  | 0 | 1 | 2 | 3 | 4 | 5 | 6 | 7 | 8 | 9 | 10 | 11 | 12 | 13 | 14 | 1997 |  |
|  | 1998 |  |  |  |  |  |  |  |  |  |  |  |  |  | 0 | 1 | 2 | 3 | 4 | 5 | 6 | 7 | 8 | 9 | 10 | 11 | 12 | 13 | 1998 |  |
|  | 1999 |  |  |  |  |  |  |  |  |  |  |  |  |  |  | 0 | 1 | 2 | 3 | 4 | 5 | 6 | 7 | 8 | 9 | 10 | 11 | 12 | 1999 |  |
|  | 2000 |  |  |  |  |  |  |  |  |  |  |  |  |  |  |  | 0 | 1 | 2 | 3 | 4 | 5 | 6 | 7 | 8 | 9 | 10 | 11 | 2000 |  |
|  | 2001 |  |  |  |  |  |  |  |  |  |  |  |  |  |  |  |  | 0 | 1 | 2 | 3 | 4 | 5 | 6 | 7 | 8 | 9 | 10 | 2001 |  |
|  | 2002 |  |  |  |  |  |  |  |  |  |  |  |  |  |  |  |  |  | 0 | 1 | 2 | 3 | 4 | 5 | 6 | 7 | 8 | 9 | 2002 |  |
|  | 2003 |  |  |  |  |  |  |  |  |  |  |  |  |  |  |  |  |  |  | 0 | 1 | 2 | 3 | 4 | 5 | 6 | 7 | 8 | 2003 |  |
|  | 2004 |  |  |  |  |  |  |  |  |  |  |  |  |  |  |  |  |  |  |  | 0 | 1 | 2 | 3 | 4 | 5 | 6 | 7 | 2004 |  |
|  | 2005 |  |  |  |  |  |  |  |  |  |  |  |  |  |  |  |  |  |  |  |  | 0 | 1 | 2 | 3 | 4 | 5 | 6 | 2005 |  |
|  | 2006 |  |  |  |  |  |  |  |  |  |  |  |  |  |  |  |  |  |  |  |  |  | 0 | 1 | 2 | 3 | 4 | 5 | 2006 |  |
|  | 2007 |  |  |  |  |  |  |  |  |  |  |  |  |  |  |  |  |  |  |  |  |  |  | 0 | 1 | 2 | 3 | 4 | 2007 |  |
|  | 2008 |  |  |  |  |  |  |  |  |  |  |  |  |  |  |  |  |  |  |  |  |  |  |  | 0 | 1 | 2 | 3 | 2008 |  |
|  | 2009 |  |  |  |  |  |  |  |  |  |  |  |  |  |  |  |  |  |  |  |  |  |  |  |  | 0 | 1 | 2 | 2009 |  |
|  | 2010 |  |  |  |  |  |  |  |  |  |  |  |  |  |  |  |  |  |  |  |  |  |  |  |  |  | 0 | 1 | 2010 |  |
|  | 2011 |  |  |  |  |  |  |  |  |  |  |  |  |  |  |  |  |  |  |  |  |  |  |  |  |  |  | 0 | 2011 |  |
|  | 2012 |  |  |  |  |  |  |  |  |  |  |  |  |  |  |  |  |  |  |  |  |  |  |  |  |  |  |  | 2012 |  |
|  | 2013 |  |  |  |  |  |  |  |  |  |  |  |  |  |  |  |  |  |  |  |  |  |  |  |  |  |  |  | 2013 |  |
|  | 2014 |  |  |  |  |  |  |  |  |  |  |  |  |  |  |  |  |  |  |  |  |  |  |  |  |  |  |  | 2014 |  |
|  | 2015 |  |  |  |  |  |  |  |  |  |  |  |  |  |  |  |  |  |  |  |  |  |  |  |  |  |  |  | 2015 |  |
|  | 2016 |  |  |  |  |  |  |  |  |  |  |  |  |  |  |  |  |  |  |  |  |  |  |  |  |  |  |  | 2016 |  |
|  | 2017 |  |  |  |  |  |  |  |  |  |  |  |  |  |  |  |  |  |  |  |  |  |  |  |  |  |  |  | 2017 |  |
|  | 2018 |  |  |  |  |  |  |  |  |  |  |  |  |  |  |  |  |  |  |  |  |  |  |  |  |  |  |  | 2018 |  |

**Supplemental Figure 2 – continued**

|  |  | **Year of follow-up** | | | | | | | | | | | | | | | | | | | | | | | | | | |  |  |
| --- | --- | --- | --- | --- | --- | --- | --- | --- | --- | --- | --- | --- | --- | --- | --- | --- | --- | --- | --- | --- | --- | --- | --- | --- | --- | --- | --- | --- | --- | --- |
|  | **J** | 1993 | 1994 | 1995 | 1996 | 1997 | 1998 | 1999 | 2000 | 2001 | 2002 | 2003 | 2004 | 2005 | 2006 | 2007 | 2008 | 2009 | 2010 | 2011 | 2012 | 2013 | 2014 | 2015 | 2016 | 2017 | 2018 | 2019 |  |  |
| **Year of diagnosis** | 1993 |  |  |  |  |  |  |  |  |  |  |  |  |  |  |  |  |  |  |  |  |  |  |  |  |  |  |  | 1993 | **Year of diagnosis** |
|  | 1994 |  |  |  |  |  |  |  |  |  |  | 0 | 1 | 2 | 3 | 4 | 5 | 6 | 7 | 8 | 9 | 10 | 11 | 12 | 13 | 14 | 15 | 16 | 1994 |  |
|  | 1995 |  |  |  |  |  |  |  |  |  |  |  | 0 | 1 | 2 | 3 | 4 | 5 | 6 | 7 | 8 | 9 | 10 | 11 | 12 | 13 | 14 | 15 | 1995 |  |
|  | 1996 |  |  |  |  |  |  |  |  |  |  |  |  | 0 | 1 | 2 | 3 | 4 | 5 | 6 | 7 | 8 | 9 | 10 | 11 | 12 | 13 | 14 | 1996 |  |
|  | 1997 |  |  |  |  |  |  |  |  |  |  |  |  |  | 0 | 1 | 2 | 3 | 4 | 5 | 6 | 7 | 8 | 9 | 10 | 11 | 12 | 13 | 1997 |  |
|  | 1998 |  |  |  |  |  |  |  |  |  |  |  |  |  |  | 0 | 1 | 2 | 3 | 4 | 5 | 6 | 7 | 8 | 9 | 10 | 11 | 12 | 1998 |  |
|  | 1999 |  |  |  |  |  |  |  |  |  |  |  |  |  |  |  | 0 | 1 | 2 | 3 | 4 | 5 | 6 | 7 | 8 | 9 | 10 | 11 | 1999 |  |
|  | 2000 |  |  |  |  |  |  |  |  |  |  |  |  |  |  |  |  | 0 | 1 | 2 | 3 | 4 | 5 | 6 | 7 | 8 | 9 | 10 | 2000 |  |
|  | 2001 |  |  |  |  |  |  |  |  |  |  |  |  |  |  |  |  |  | 0 | 1 | 2 | 3 | 4 | 5 | 6 | 7 | 8 | 9 | 2001 |  |
|  | 2002 |  |  |  |  |  |  |  |  |  |  |  |  |  |  |  |  |  |  | 0 | 1 | 2 | 3 | 4 | 5 | 6 | 7 | 8 | 2002 |  |
|  | 2003 |  |  |  |  |  |  |  |  |  |  |  |  |  |  |  |  |  |  |  | 0 | 1 | 2 | 3 | 4 | 5 | 6 | 7 | 2003 |  |
|  | 2004 |  |  |  |  |  |  |  |  |  |  |  |  |  |  |  |  |  |  |  |  | 0 | 1 | 2 | 3 | 4 | 5 | 6 | 2004 |  |
|  | 2005 |  |  |  |  |  |  |  |  |  |  |  |  |  |  |  |  |  |  |  |  |  | 0 | 1 | 2 | 3 | 4 | 5 | 2005 |  |
|  | 2006 |  |  |  |  |  |  |  |  |  |  |  |  |  |  |  |  |  |  |  |  |  |  | 0 | 1 | 2 | 3 | 4 | 2006 |  |
|  | 2007 |  |  |  |  |  |  |  |  |  |  |  |  |  |  |  |  |  |  |  |  |  |  |  | 0 | 1 | 2 | 3 | 2007 |  |
|  | 2008 |  |  |  |  |  |  |  |  |  |  |  |  |  |  |  |  |  |  |  |  |  |  |  |  | 0 | 1 | 2 | 2008 |  |
|  | 2009 |  |  |  |  |  |  |  |  |  |  |  |  |  |  |  |  |  |  |  |  |  |  |  |  |  | 0 | 1 | 2009 |  |
|  | 2010 |  |  |  |  |  |  |  |  |  |  |  |  |  |  |  |  |  |  |  |  |  |  |  |  |  |  | 0 | 2010 |  |
|  | 2011 |  |  |  |  |  |  |  |  |  |  |  |  |  |  |  |  |  |  |  |  |  |  |  |  |  |  |  | 2011 |  |
|  | 2012 |  |  |  |  |  |  |  |  |  |  |  |  |  |  |  |  |  |  |  |  |  |  |  |  |  |  |  | 2012 |  |
|  | 2013 |  |  |  |  |  |  |  |  |  |  |  |  |  |  |  |  |  |  |  |  |  |  |  |  |  |  |  | 2013 |  |
|  | 2014 |  |  |  |  |  |  |  |  |  |  |  |  |  |  |  |  |  |  |  |  |  |  |  |  |  |  |  | 2014 |  |
|  | 2015 |  |  |  |  |  |  |  |  |  |  |  |  |  |  |  |  |  |  |  |  |  |  |  |  |  |  |  | 2015 |  |
|  | 2016 |  |  |  |  |  |  |  |  |  |  |  |  |  |  |  |  |  |  |  |  |  |  |  |  |  |  |  | 2016 |  |
|  | 2017 |  |  |  |  |  |  |  |  |  |  |  |  |  |  |  |  |  |  |  |  |  |  |  |  |  |  |  | 2017 |  |
|  | 2018 |  |  |  |  |  |  |  |  |  |  |  |  |  |  |  |  |  |  |  |  |  |  |  |  |  |  |  | 2018 |  |

**Supplemental Figure 2 – continued**

|  |  | **Year of follow-up** | | | | | | | | | | | | | | | | | | | | | | | | | | |  |  |
| --- | --- | --- | --- | --- | --- | --- | --- | --- | --- | --- | --- | --- | --- | --- | --- | --- | --- | --- | --- | --- | --- | --- | --- | --- | --- | --- | --- | --- | --- | --- |
|  | **K** | 1993 | 1994 | 1995 | 1996 | 1997 | 1998 | 1999 | 2000 | 2001 | 2002 | 2003 | 2004 | 2005 | 2006 | 2007 | 2008 | 2009 | 2010 | 2011 | 2012 | 2013 | 2014 | 2015 | 2016 | 2017 | 2018 | 2019 |  |  |
| **Year of diagnosis** | 1993 |  |  |  |  |  |  |  |  |  |  | 0 | 1 | 2 | 3 | 4 | 5 | 6 | 7 | 8 | 9 | 10 | 11 | 12 | 13 | 14 | 15 | 16 | 1993 | **Year of diagnosis** |
|  | 1994 |  |  |  |  |  |  |  |  |  |  |  | 0 | 1 | 2 | 3 | 4 | 5 | 6 | 7 | 8 | 9 | 10 | 11 | 12 | 13 | 14 | 15 | 1994 |  |
|  | 1995 |  |  |  |  |  |  |  |  |  |  |  |  | 0 | 1 | 2 | 3 | 4 | 5 | 6 | 7 | 8 | 9 | 10 | 11 | 12 | 13 | 14 | 1995 |  |
|  | 1996 |  |  |  |  |  |  |  |  |  |  |  |  |  | 0 | 1 | 2 | 3 | 4 | 5 | 6 | 7 | 8 | 9 | 10 | 11 | 12 | 13 | 1996 |  |
|  | 1997 |  |  |  |  |  |  |  |  |  |  |  |  |  |  | 0 | 1 | 2 | 3 | 4 | 5 | 6 | 7 | 8 | 9 | 10 | 11 | 12 | 1997 |  |
|  | 1998 |  |  |  |  |  |  |  |  |  |  |  |  |  |  |  | 0 | 1 | 2 | 3 | 4 | 5 | 6 | 7 | 8 | 9 | 10 | 11 | 1998 |  |
|  | 1999 |  |  |  |  |  |  |  |  |  |  |  |  |  |  |  |  | 0 | 1 | 2 | 3 | 4 | 5 | 6 | 7 | 8 | 9 | 10 | 1999 |  |
|  | 2000 |  |  |  |  |  |  |  |  |  |  |  |  |  |  |  |  |  | 0 | 1 | 2 | 3 | 4 | 5 | 6 | 7 | 8 | 9 | 2000 |  |
|  | 2001 |  |  |  |  |  |  |  |  |  |  |  |  |  |  |  |  |  |  | 0 | 1 | 2 | 3 | 4 | 5 | 6 | 7 | 8 | 2001 |  |
|  | 2002 |  |  |  |  |  |  |  |  |  |  |  |  |  |  |  |  |  |  |  | 0 | 1 | 2 | 3 | 4 | 5 | 6 | 7 | 2002 |  |
|  | 2003 |  |  |  |  |  |  |  |  |  |  |  |  |  |  |  |  |  |  |  |  | 0 | 1 | 2 | 3 | 4 | 5 | 6 | 2003 |  |
|  | 2004 |  |  |  |  |  |  |  |  |  |  |  |  |  |  |  |  |  |  |  |  |  | 0 | 1 | 2 | 3 | 4 | 5 | 2004 |  |
|  | 2005 |  |  |  |  |  |  |  |  |  |  |  |  |  |  |  |  |  |  |  |  |  |  | 0 | 1 | 2 | 3 | 4 | 2005 |  |
|  | 2006 |  |  |  |  |  |  |  |  |  |  |  |  |  |  |  |  |  |  |  |  |  |  |  | 0 | 1 | 2 | 3 | 2006 |  |
|  | 2007 |  |  |  |  |  |  |  |  |  |  |  |  |  |  |  |  |  |  |  |  |  |  |  |  | 0 | 1 | 2 | 2007 |  |
|  | 2008 |  |  |  |  |  |  |  |  |  |  |  |  |  |  |  |  |  |  |  |  |  |  |  |  |  | 0 | 1 | 2008 |  |
|  | 2009 |  |  |  |  |  |  |  |  |  |  |  |  |  |  |  |  |  |  |  |  |  |  |  |  |  |  | 0 | 2009 |  |
|  | 2010 |  |  |  |  |  |  |  |  |  |  |  |  |  |  |  |  |  |  |  |  |  |  |  |  |  |  |  | 2010 |  |
|  | 2011 |  |  |  |  |  |  |  |  |  |  |  |  |  |  |  |  |  |  |  |  |  |  |  |  |  |  |  | 2011 |  |
|  | 2012 |  |  |  |  |  |  |  |  |  |  |  |  |  |  |  |  |  |  |  |  |  |  |  |  |  |  |  | 2012 |  |
|  | 2013 |  |  |  |  |  |  |  |  |  |  |  |  |  |  |  |  |  |  |  |  |  |  |  |  |  |  |  | 2013 |  |
|  | 2014 |  |  |  |  |  |  |  |  |  |  |  |  |  |  |  |  |  |  |  |  |  |  |  |  |  |  |  | 2014 |  |
|  | 2015 |  |  |  |  |  |  |  |  |  |  |  |  |  |  |  |  |  |  |  |  |  |  |  |  |  |  |  | 2015 |  |
|  | 2016 |  |  |  |  |  |  |  |  |  |  |  |  |  |  |  |  |  |  |  |  |  |  |  |  |  |  |  | 2016 |  |
|  | 2017 |  |  |  |  |  |  |  |  |  |  |  |  |  |  |  |  |  |  |  |  |  |  |  |  |  |  |  | 2017 |  |
|  | 2018 |  |  |  |  |  |  |  |  |  |  |  |  |  |  |  |  |  |  |  |  |  |  |  |  |  |  |  | 2018 |  |

**Supplemental Table S1**

| **Supplemental Table S1.** Period of diagnosis considered for the calculation of five-year relative survival at diagnosis and the conditional 5-year relative survival at one through ten years post-diagnosis. | | | | | |  |
| --- | --- | --- | --- | --- | --- | --- |
|  |  |  |  |  |  |  |
|  | **Years of diagnosis** | **Period of diagnosis** | | | **Graphical illustration of the survival data used** |  |
| **5-year relative survival from *x* years after diagnosis** | 0 | 2003 | - | 2018 | Supplemental Figure 2A |  |
|  | 1 | 2002 | - | 2018 | Supplemental Figure 2B |  |
|  | 2 | 2001 | - | 2017 | Supplemental Figure 2C |  |
|  | 3 | 2000 | - | 2016 | Supplemental Figure 2D |  |
|  | 4 | 1999 | - | 2015 | Supplemental Figure 2E |  |
|  | 5 | 1998 | - | 2014 | Supplemental Figure 2F |  |
|  | 6 | 1997 | - | 2013 | Supplemental Figure 2G |  |
|  | 7 | 1996 | - | 2012 | Supplemental Figure 2H |  |
|  | 8 | 1995 | - | 2011 | Supplemental Figure 2I |  |
|  | 9 | 1994 | - | 2010 | Supplemental Figure 2J |  |
|  | 10 | 1993 | - | 2009 | Supplemental Figure 2K |  |

**Supplemental references**

1. Brenner H, Rachet B. Hybrid analysis for up-to-date long-term survival rates in cancer registries with delayed recording of incident cases. Eur J Cancer. 2004;40(16):2494-501.

2. Brenner H, Gefeller O. Deriving more up-to-date estimates of long-term patient survival. J Clin Epidemiol. 1997;50(2):211-6.

3. Dickman PW, Adami HO. Interpreting trends in cancer patient survival. J Intern Med. 2006;260(2):103-17.
